# Single-dose botulinum toxin type a compared with repeated-dose for treatment of trigeminal neuralgia: a pilot study

**DOI:** 10.1186/s10194-017-0793-3

**Published:** 2017-08-10

**Authors:** Haifeng Zhang, Yajun Lian, Nanchang Xie, Chen Chen, Yake Zheng

**Affiliations:** Department of Neurology, First Affiliated Hospital of Zhengzhou University, 1 Jianshe East R, Zhengzhou City, HeNan Province 450052 People’s Republic of China

**Keywords:** Botulinum toxin type a, Trigeminal neuralgia, Single dosing, Repeated dosing

## Abstract

**Background:**

Several RCT studies including ours, seem to prove the role of Botulinum toxin type A (BTX-A) in the treatment of trigeminal neuralgia (TN), but no standardized dosing regimen has been established. In our study, we compare two different methods of administration: single-dose or repeated-dose strategy which was most frequently applied over the years in our centre.

**Methods:**

An open-label trail was conducted. One hundred patients with classic TN symptoms were recruited, and randomly and equally apportioned to single- or repeated-dose group. Patients in the single-dose group received a local BTX-A injection of 70 to 100 U. The repeated-dose group received an initial BTX-A injection of 50 to 70 U and then another of equal volume 2 weeks later. All patients were followed for 6 months.

**Results:**

In the single- and repeated-dose groups, 44 and 37, respectively, completed the entire study. The groups were statistically similar in TN frequency, time between treatment and effect, time to peak effect, VAS scores, and rates of adverse reactions (latency and duration). However, the single-dose group experienced significantly longer duration of effect (*P* = 0.032).

**Conclusions:**

The single- and repeated-dosing BTX-A regimens were largely comparable in efficacy and safety. This study suggests that repeated dosing has no advantage over single dosing of BTX-A for TN. Dosing should be adjusted for the individual patient.

## Background

Trigeminal neuralgia (TN) is severe and recurring pain distributed unilaterally along a branch of the trigeminal nerve. [1, 2] TN can be triggered by brushing the teeth, washing the face, drinking liquids, or shaving. Patients become fearful of performing these life activities, in anticipation of long-lasting stabbing pain. TN patients may display a haggard facial expression and depressive mood, as their quality of life and ability to work is compromised. The most common treatment is anti-epileptics such as carbamazepine and oxcarbazepine. However, these medications may have to be discontinued due to intolerable side effects. Neurosurgical interventions remain debatable for safety and efficacy concerns [3].

Botulinum toxin type A (BTX-A) is an exotoxin released by the gram-positive bacterium *Clostridium botulinum*. Its initial medicinal use was in the management of blepharospasm and hemifacial spasm [4]. In recent years, BTX-A has been used for the relief of chronic migraine and many other types of headache [5–8]. The application of BTX-A to relieve TN was first reported in 2002, and its safety and effectiveness was later confirmed by series studies [9–19]. These findings suggest that local intradermal or submucosal injections of BTX-A may be a promising therapy for TN.

In 2009, our group initiated a study of TN treatment using BTX-A, and a clinical database was established. Through several clinical trials, our group has shown that BTX-A can provide long-lasting relief of TN symptoms, with only mild or moderate side effects [10, 16–18]. However, before clinical application of BTX-A for TN treatment can become routine, a standardized therapeutic regimen should be established. In particular, there is no consensus regarding dosage or treatment schedule, and relevant clinical trial data are scarce. In a randomized, double blind, placebo-controlled study in 2014, we reported that doses of 25 U or 75 U BTX-A were similar in short-term efficacy for treatment of TN [17]. Since the efficacy could not be improved by simply increasing the dose, we asked whether the efficacy may change if the dosing time was extended. To answer this question, for the present study we conducted an open-label trial to compare the efficacy, safety, and tolerability of single or repeated administrations of BTX-A in patients with TN.

## Methods

### Study design

This is an open-label trial with an intended sample size of 100 patients. The trial was approved by the local ethics committee. Before treatment, each patient was counseled regarding the goal, procedure, and possible adverse reactions, and they were informed of the risk of transient attendant weakness and disfigurement produced by localized administration of BTX-A.

The patients were randomized into a single-dose or a repeated-dose group (*n* = 50, each). Patients in the single-dose group received a local BTX-A (70–100 U) injection at the site of pain. Patients assigned to the repeated-dose group received the initial treatment of BTX-A and then another injection 2 weeks later, with 50–70 U per time and a total dosage of 100–140 U. All the patients were followed for 6 months, and they had the freedom to withdraw from the study at any time. Patients who completed the entire 6-month follow-up were eligible for the final study analysis.

### Study participants

All the medical information from October 2012 to June 2015 was collected from the Department of Neurology, First Affiliated Hospital of Zhengzhou University. All the recruited patients had primary TN and received BTX-A treatment. The criteria for inclusion in the study were the following: underwent comprehensive physical examination and medical tests to exclude the existence of coagulopathy, or severe heart, liver, kidney, or other organ dysfunction; underwent coronal magnetic resonance imaging or computed tomography to rule out secondary TN; diagnosis of classical TN based on the current version of the International Classification of Headache Disorders (ICHD-II) [20]; baseline TN-associated VAS score ≥ 6; and analgesics received before the study remained unchanged during the course of the study.

Patients with any of the following were excluded from the study: prior BTX-A treatment; comorbid diseases that may be exacerbated by BTX-A (e.g., myasthenia gravis, motor neuron disease, or Lambert-Eaton syndrome); infection or dermatosis at the injection site; receiving drugs with neuromuscular junction toxicity 1 week before BTX-A treatment (e.g. quinine, aminoglycosides or penicillamine); unstable psychiatric disease; history of drug abuse or addiction; pregnant, plans for pregnancy, or lactating; analgesic therapies begun at any time during the study.

### Treatment

BTX-A (100 U of *Clostridium botulinum* type A neurotoxin complex, 5 mg gelatin, 25 mg dextran, and 25 mg saccharose) was obtained from Lanzhou Biological Products Institute (Lanzhou, China). All treatments were administered in a specially-designated treatment room, which is equipped with facilities to ensure safety in the event of severe reaction or emergency. During injections, the patients lay supine on a bed.

The BTX-A was dissolved in 2 mL of saline to 50 U/mL before use. The injections were administered intradermally, submucosally, or both, at the site of pain using a 1-mL syringe with a 0.45 × 16 mm needle. The injection depth was nearly 0.1 cm, and, for multiple site injection, 15-mm distance was set between injection sites. There were 15–25 injection sites in total, with 1.25–5 U at each site. For single injection patients, the total dosage of BTX-A were set within 70–100 U. While for patients receiving two times of BTX-A, the dosage was within 50–70 U each time.

### Follow up and efficacy assessment

The patients were required to provide a daily diary of their pain symptoms, including provoking factors, frequency of TN attacks, severity of pain (according to an 11-point visual analogue scale, VAS) and adverse reaction. Prior to the first injection, patient demographics, gender, age, presence of trigger zone, side of involvement, and the nerve branch involved were also recorded. Follow-up visits were made every week during the first month after the injection, and once per month thereafter.

To determine the efficacy of treatment, during each follow-up visit the pain severity and attack frequency from baseline to endpoint, time until the drug treatment took effect, and the peak time and duration of drug efficacy were noted. A drug response was defined as ≥50% reduction in mean pain score from baseline to endpoint.

### Safety assessment

Safety was measured according to the occurrence of adverse events, and recorded with date of onset, severity, duration, treatment required (if any), and outcome.

### Statistical analysis

The quantitative data were expressed as mean ± standard deviation. Differences between independent groups were evaluated by means of a *t*-test. The chi-squared test was performed to assess differences in treatment responses. The data were analyzed using SPSS20.0 software. *P* < 0.05 was considered a significant statistical difference.

## Results

### Patient disposition

After the 6-month follow-up period, 44 and 37 patients in the single-dose and repeated-dose groups, respectively, had completed the entire study (Table 1). Nineteen patients loss due to patient drop out (The agreement stipulates that they may withdraw at any time) or incomplete follow up. The groups were well matched at baseline for age, gender, duration of symptoms, and side and nerve branch involved (*P >* 0.05).

**Table 1:** Baseline demographic characteristics and clinical data of TN patients given single or repeated dose BTX-A^a^

		Single dose	Repeated dose	*P*
Subjects, n		44	37	
Age, y^b^		60.73 ± 10.88	57.14 ± 10.39	0.056
Disease course, mo ^b^		73.50 ± 63.63	50.46 ± 43.95	0.059
Gender	Male	19 (43.2%)	16 (43.2%)	1
	Female	25 (56.8%)	21 (56.8%)	
Side	Left	17 (38.6%)	14 (37.8%)	1
	Right	27 (61.4%)	23 (62.2%)	
Nerve branch	1	5 (11.4%)	3 (8.1%)	0.324
	2	10 (22.7%)	11 (29.7%)	
	3	11 (25.0%)	8 (21.6%)	
	1, 2	8 (18.2%)	3 (8.1%)	
	1, 3	2 (4.5%)	0 (0.0%)	
	2, 3	8 (18.2%)	10 (27.0%)	
	1, 2, 3	0 (0.0%)	2 (5.4%)	
Toothache	Yes	11 (25.0%)	15 (40.5%)	0.158
	No	33 (75.0%)	22 (59.5%)	

^a^Reported as n (%), unless indicated otherwise ^b^ y = year, mo = month

### Efficacy results

To investigate differences in treatment efficacy between the single- and repeated-dose groups, the rate of TN occurrence was determined before and after each dosing (Table 2); results from the *t*-test indicated no significant difference between the single- and repeated-dose groups (*P* > 0.05). The times to drug effect and peak efficacy times of the 2 groups were also statistically similar. However, the duration of efficacy in the single-dose group was significantly longer than that of the repeated-dose group (*P* = 0.032).

**Table 2:** Evaluation on the efficacy of single and repeated doses of BTX-A

		Single dose	Repeated dose	*P* value
Subjects, *n*		44	37	
TN frequency, *n*	Before treatment	18.40 ± 24.89	22.85 ± 28.26	0.454
	After treatment	2.07 ± 3.62	3.19 ± 9.23	0.461
Time to take effect, d		8.51 ± 6.37	10.08 ± 6.63	0.282
Time to peak efficacy, d		30.86 ± 18.90	25.62 ± 16.25	0.189
Efficacy maintenance, mo		4.76 ± 1.73	3.87 ± 1.92	0.032

*d* day, *mo* month

The drug response rates of the single- and repeated-dose groups (i.e., percent of patients with ≥50% reduction in mean pain score from baseline to endpoint) were not significantly different (*P >* 0.05, Table 3)

**Table 3:** Response to treatment at follow-ups throughout the study, *n* (%)

	Single dose	Repeated dose	*P*
Subjects, n	44	37	
1 mo	35 (79.5)	31 (83.8)	0.202
2 mo	40 (90.9)	31 (83.8)	0.168
3 mo	40 (90.9)	31 (83.8)	0.168
4 mo	35 (79.5)	24 (64.9)	0.068
5 mo	31 (70.5)	23 (62.2)	0.137
6 mo	27 (61.4)	19 (51.4)	0.119

*mo* month

### VAS score

**Fig. 1:**
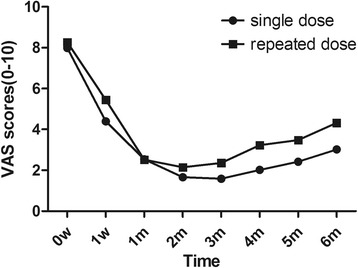
Monthly VAS scores of TN patients. Patients in the single- and repeated-dose groups were followed for 6 consecutive months after the first BTX-A injection. At each follow-up, the VAS scores of the 2 groups were comparable

At baseline, each group was well matched for severity of VAS scores (8.26 ± 1.68 and 7.98 ± 1.60 for the single- and repeated-dose groups, respectively; Table 4). At each assessment after the BTX-A injection, the VAS scores of the 2 groups were similar (*P >* 0.05, Fig. 1). These results suggest that, throughout the study, there was no significant difference in the efficacy between the 2 dosing modalities (*P* > 0.05).

**Table 4:** VAS scores at baseline and during the 6-month follow-up

Time	Dose group	VAS	*P*
0w	Single	7.99 ± 1.60	0.441
	Repeated	8.27 ± 1.69	
1w	Single	4.40 ± 2.21	0.089
	Repeated	5.45 ± 3.08	
1mo	Single	2.54 ± 2.37	0.977
	Repeated	2.52 ± 2.72	
2mo	Single	1.66 ± 2.11	0.374
	Repeated	2.15 ± 2.69	
3mo	Single	1.59 ± 2.17	0.198
	Repeated	2.36 ± 3.01	
4mo	Single	2.02 ± 2.53	0.084
	Repeated	3.23 ± 3.49	
5mo	Single	2.42 ± 2.84	0.150
	Repeated	3.48 ± 3.58	
6mo	Single	3.02 ± 3.29	0.095
	Repeated	4.32 ± 3.61	

*w* week, *mo* month

Single dose, *n* = 44; repeated dose, *n* = 37

### Safety assessment

Adverse reactions of the patients in the 2 groups were mild to moderate, and therefore the safety profiles of both regimens were considered satisfactory (Table 5). Rates of adverse reactions of the 2 groups were similar, both in duration and time to the appearance of these side effects (*P >* 0.05).

**Table 5:** Adverse reactions associated with BTX-A treatment

	Single dose	Repeated dose	*P*
Subjects, *n*	44	37	
Patients experiencing side effects, *n* (%)	7 (15.9)	7 (18.9)	0.774
Time before side effects, d	2.22 ± 7.2042	2.405 ± 5.7805	0.904
Duration of side effects, d	7.068 ± 20.9411	8.703 ± 22.0893	0.734

*d* day

## Discussion

BTX-A is a *Clostridium botulinum*-derived exotoxin that has been used widely in cosmetology and for treating dysmyotonia [16]. Recent years have seen an enormous interest in the application of BTX-A to relieve chronic migraine and other types of headache [5–8]. In addition, BTX-A has been used for neuropathic pain, including post-herpetic neuralgia [21], diabetic neuropathic pain [22, 23], and occipital neuralgia [24]. However, to date, no standardized dosing regimen has been established. We conducted this pilot study to evaluate the efficacy and safety of single-dose BTX-A compared with repeated-dose BTX-A in TN. We found during a 6-month follow-up that single and repeated dosing of BTX-A in TN patients have comparable efficacy and side effects.

Since 2002, there have been many reports concerning BTX-A treatment for TN [9–19]. Several randomized clinical studies have focused on efficacy and safety issues [25]. These findings have triggered much interest in clarifying questions such as the optimal dose, duration of therapeutic efficacy, and common adverse reactions [26]. Our group has also been working on determining the efficacy and safety of the clinical use of BTX-A [10, 16–18].

The impetus for this study was our clinical experience that, in some TN patients showing poor responses to BTX-A, repeated dosing may lead to better pain relief. In an earlier 14-month follow-up study involving 88 patients with TN, we found that the duration of therapeutic effect (i.e., reduction in VAS scores) may be due mostly to the first injection of BTX-A [16]. Therefore, we recommended a relatively low dose at the first injection, and, if necessary, an additional 1-to-3 doses to treat intractable TN, 2-to-4 weeks later.

However, we still wondered if repeated dosing conferred a therapeutic advantage over a single dose. Thus, we determined to perform the present pilot open-label trial to compare the efficacy and safety profiles of single or repeated doses of BTX-A. An important factor in our study design was that a medium dose (50–100 U) of BTX-A was used, because this is within the most common dosing range in our clinical practice. Moreover, a relatively larger dose was used in patients who received the repeated dose (100–140 U compared to 70–100 U in single dose group). Although our previous study indicated that such a difference in dosage may not influence efficacy and safety, at least in the short term, future studies are warranted to determine doses for single and repeated dosing regimens.

Our present study suggests that both the single and repeated dosing regimens were similar in frequency rates and severity of side effects. Thus, it is reasonable to suggest that the occurrence of adverse reactions is more relevant to the injection procedure. This may require more attention in future practice. For example, the orbicular muscle of the mouth should be avoided, and a reduced dose may be required at each injection site (e.g., 1.25–2.5 U/point).

The results of our study showed that repeated dosing of BTX-A did not contribute to improved clinical outcome in TN patients. Since repeated dosing is accompanied by increased cost and inconvenience, single dosing may be the best choice in management of TN. Nevertheless, for patients who respond poorly to the first injection, a second dosing may still be safely performed. Given the inherent limitation of this pilot study, a future study is anticipated to provide more comprehensive evaluation of the optimal BTX-A regimen for clinical management of TN.

## Conclusions

The single- and repeated-dosing BTX-A regimens were largely comparable in efficacy and safety. This study suggests that repeated dosing has no advantage over single dosing of BTX-A for TN. Dosing should be adjusted for the individual patient.

### Highlight

1. The single- and repeated-dosing of BTX-A were comparable in efficacy and safety in TN.

2. Repeated dosing has no advantage over single dosing of BTX-A for TN.

## References

[1] Burchiel KJ (2003). A new classification for facial pain. Neurosurgery.

[2] Cruccu G, Gronseth G, Alksne J (2008). AAN-EFNS guidelines on trigeminal neuralgia management. Eur J Neurol.

[3] Love S, Coakham HB (2001). Trigeminal neuralgia: pathology and pathogenesis. Brain.

[4] Wu CJ, Shen JH, Chen Y, Lian YJ (2011). Comparison of two different formulations of botulinum toxin a for the treatment of blepharospasm and hemifacial spasm. Turk Neurosurg.

[5] Aurora SK, Dodick DW, Turkel CC (2010). OnabotulinumtoxinA for treatment of chronic migraine: results from the double-blind, randomized, placebo-controlled phase of the PREEMPT 1 trial. Cephalalgia.

[6] Diener HC, Dodick DW, Aurora SK (2010). OnabotulinumtoxinA for treatment of chronic migraine: results from the double-blind, randomized, placebo-controlled phase of the PREEMPT 2 trial. Cephalalgia.

[7] Negro A, Curto M, Lionetto L, Giamberardino MA, Martelletti P (2016). Chronic migraine treatment: from OnabotulinumtoxinA onwards. Expert Rev Neurother.

[8] Sandrini G, De Icco R, Tassorelli C, Smania N, Tamburin S (2017). Botulinum neurotoxin type a for the treatment of pain: not just in migraine and trigeminal neuralgia. J Headache Pain..

[9] Micheli F, Scorticati MC, Raina G (2002). Beneficial effects of botulinum toxin type a for patients with painful tic convulsif. Clin Neuropharmacol.

[10] Wu CJ, Lian YJ, Zheng YK (2012). Botulinum toxin type a for the treatment of trigeminal neuralgia: results from a randomized, double-blind, placebo-controlled trial. Cephalalgia.

[11] Allam N, Brasil-Neto JP, Brown G, Tomaz C (2005). Injections of botulinum toxin type a produce pain alleviation in intractable trigeminal neuralgia. Clin J Pain.

[12] Ngeow WC, Nair R (2010). Injection of botulinum toxin type a (BOTOX) into trigger zone of trigeminal neuralgia as a means to control pain. Oral Surg Oral Med Oral Pathol Oral Radiol Endod.

[13] Piovesan EJ, Leite LS, Teive HG (2011). Botulinum toxin type-a effect as a preemptive treatment in a model of acute trigeminal pain: a pre-clinical double-blind and placebo-controlled study. Arq Neuropsiquiatr.

[14] Piovesan EJ, Teive HG, Kowacs PA, Della CMV, Werneck LC, Silberstein SD (2005). An open study of botulinum-a toxin treatment of trigeminal neuralgia. Neurology.

[15] Türk U, Ilhan S, Alp R, Sur H (2005). Botulinum toxin and intractable trigeminal neuralgia. Clin Neuropharmacol.

[16] Li S, Lian YJ, Chen Y (2014). Therapeutic effect of Botulinum toxin-a in 88 patients with trigeminal neuralgia with 14-month follow-up. J Headache Pain..

[17] Zhang H, Lian Y, Ma Y (2014). Two doses of botulinum toxin type a for the treatment of trigeminal neuralgia: observation of therapeutic effect from a randomized, double-blind, placebo-controlled trial. J Headache Pain..

[18] Xia JH, He CH, Zhang HF (2016). Botulinum toxin a in the treatment of trigeminal neuralgia. Int J Neurosci.

[19] Morra ME, Elgebaly A, Elmaraezy A (2016). Therapeutic efficacy and safety of Botulinum toxin a therapy in trigeminal neuralgia: a systematic review and meta-analysis of randomized controlled trials. J Headache Pain..

[20] Olesen J, Steiner TJ (2004). The international classification of headache disorders, 2nd edn (ICDH-II). J Neurol Neurosurg Psychiatry.

[21] Apalla Z, Sotiriou E, Lallas A, Lazaridou E, Ioannides D (2013). Botulinum toxin a in postherpetic neuralgia: a parallel, randomized, double-blind, single-dose, placebo-controlled trial. Clin J Pain.

[22] Chen WT, Yuan RY, Chiang SC (2013). OnabotulinumtoxinA improves tactile and mechanical pain perception in painful diabetic polyneuropathy. Clin J Pain.

[23] Yuan RY, Sheu JJ, Yu JM (2009). Botulinum toxin for diabetic neuropathic pain: a randomized double-blind crossover trial. Neurology.

[24] Taylor M, Silva S, Cottrell C (2008). Botulinum toxin type-a (BOTOX) in the treatment of occipital neuralgia: a pilot study. Headache.

[25] Kowacs PA, Utiumi MA, Nascimento FA, Piovesan EJ, Teive HA (2015). OnabotulinumtoxinA for trigeminal neuralgia: a review of the available data. Arq Neuropsiquiatr.

[26] Shehata HS, El-Tamawy MS, Shalaby NM, Ramzy G (2013). Botulinum toxin-type a: could it be an effective treatment option in intractable trigeminal neuralgia. J Headache Pain.

